# Landscape transformations produce favorable roosting conditions for turkey vultures and black vultures

**DOI:** 10.1038/s41598-021-94045-3

**Published:** 2021-07-20

**Authors:** Jacob E. Hill, Kenneth F. Kellner, Bryan M. Kluever, Michael L. Avery, John S. Humphrey, Eric A. Tillman, Travis L. DeVault, Jerrold L. Belant

**Affiliations:** 1 Global Wildlife Conservation Center, State University of New York College of Environmental Science and Forestry, Syracuse, NY 13210 USA; 2 Savannah River Ecology Laboratory, University of Georgia, Aiken, SC 29802 USA; 3 United States Department of Agriculture, Wildlife Services, National Wildlife Research Center, Florida Field Station, Gainesville, FL 32641 USA

**Keywords:** Conservation biology, Urban ecology

## Abstract

Recent increases in turkey vulture (*Cathartes aura*) and black vulture (*Coragyps atratus*) populations in North America have been attributed in part to their success adapting to human-modified landscapes. However, the capacity for such landscapes to generate favorable roosting conditions for these species has not been thoroughly investigated. We assessed the role of anthropogenic and natural landscape elements on roosting habitat selection of 11 black and 7 turkey vultures in coastal South Carolina, USA using a GPS satellite transmitter dataset derived from previous research. Our dataset spanned 2006–2012 and contained data from 7916 nights of roosting. Landscape fragmentation, as measured by land cover richness, influenced roosting probability for both species in all seasons, showing either a positive relationship or peaking at intermediate values. Roosting probability of turkey vultures was maximized at intermediate road densities in three of four seasons, and black vultures showed a positive relationship with roads in fall, but no relationship throughout the rest of the year. Roosting probability of both species declined with increasing high density urban cover throughout most of the year. We suggest that landscape transformations lead to favorable roosting conditions for turkey vultures and black vultures, which has likely contributed to their recent proliferations across much of the Western Hemisphere.

## Introduction

More than three quarters of Earth’s terrestrial surface is impacted by the presence of humans^1^. Since the industrial era, buildings and paved land have collectively increased to a global expanse exceeding 2.47 million km^2^, while total forest and wetland cover concurrently declined by 30% and 55%, respectively^2,3^. Converting land to suit the needs of humans not only reduces natural habitat, but often fragments the remaining habitat into isolated patches that exist within a matrix of human development^4^.

Land conversions have been detrimental to many bird species due to factors such as reduced resource availability, the spread of invasive species, and increased human-wildlife conflict^5^. As a result, nearly one quarter of the global bird population has been lost since the advent of agriculture^6^. Some species, however, have been able to thrive across landscapes modified by humans. Human presence can result in novel food items that birds exploit^7^, and in structures like buildings or communication towers that can serve as suitable nesting or roosting sites^8^. Increases in edge habitat resulting from habitat fragmentation may be advantageous for edge specialists, and some species can exploit resources provided by agricultural landscapes^9,10^.

Two avian species that have seemingly benefited from human landscape modifications are turkey vultures (*Cathartes aura*) and black vultures (*Coragyps atratus*). Between 1966 and 2015, population indexes for these species in the United States increased each year by 2.19% and 4.77% on average, respectively^11^. Concurrently, the geographic ranges of both species across North and South America have also expanded dramatically^12–14^. These trends pose a marked contrast to vulture populations in other parts of the world, many of which have declined precipitously over the past few decades^15^. Compared to species on other continents, turkey vultures and black vultures have a better conservation status due in part to ecological traits such as faster life-history strategies^15^. Additionally, they are to exposed to more favorable social and political conditions including greater legal protections and decreased exposure to intentional and incidental poisoning, which collectively contribute to an improved conservation status^12,15^.

In addition to direct conservation actions, black and turkey vultures also may have incidentally benefited from human development and land use changes. At night when they are inactive, vultures often roost communally in groups that may exceed several hundred individuals e.g.^16^ and human development may lead to favorable conditions for vulture roosting. Two factors hypothesized to influence vulture roosting are air currents and distance to food sources^17–20^. Roads may provide both of these features with carrion produced as the result of vehicle collisions and thermal currents emanating from the paved surface^21–23^. Similarly, urban areas may contribute food from garbage while providing thermal currents from impervious surface cover^24,25^. Additionally, fragmented landscapes could be beneficial by combining these attributes from developed land with open areas that facilitate detection of carrion^21^. The interfaces of open areas and forests occurring as a result of habitat fragmentation may facilitate flight through obstruction currents, produced when wind strikes the tree line^18,26,27^. Furthermore, landscape fragmentation results in contrasting surface temperatures, which produces strong thermal currents that vultures heavily use for flight^23^.

In addition to these anthropogenic features of the landscape, natural elements also influence roost site selection. Vultures sometimes select forests and woody wetlands for roosting due to availability of perching sites and favorable thermal conditions^18,28^. Roosts at greater elevations may be preferred because they produce greater uplift, which aids in flight^18^. Both species may roost near water bodies because they provide opportunities for drinking and bathing, and large trees that serve as roosts tend to be located close to water^18,19^. The comparative influence of natural and anthropogenic landscape elements on roost site selection could also be temporally dynamic because vultures have larger home ranges in warmer months due to more suitable flight conditions and greater competition for carrion^29^. Examining the relationship between vulture roosting and landscape attributes can lend insight into the favorability of environmental alterations for vultures and elucidate ecological factors contributing to their range expansions. Furthermore, large aggregations of vultures are often a source of conflict with humans e.g.^30–34^. A better understanding of factors influencing roost site selection can therefore be used to predict where conflict with humans is likely to occur and aid in mitigation efforts.

Using a GPS satellite transmitter dataset derived from previous research, we tested the hypothesis that anthropogenic landscape alterations influence nighttime roosting locations of black and turkey vultures. We predicted that vultures would be more likely to roost closer to roads, closer to water, and at higher elevations. We also predicted that roosting would be positively associated with urban land cover. Lastly, we predicted that vulture roosting would be more likely to occur in areas with increased landscape fragmentation. We tested our predictions separately for each of the four seasons to account for seasonal variation in movement that could potentially affect roost site selection.

## Methods

### Data collection

We conducted this study in and around Marine Corps Air Station (MCAS) Beaufort (32.4735° N. 80.7194° W), about 5 km from downtown Beaufort, South Carolina, USA. Beaufort has an area of 65.5 km^2^ and population of approximately 13,000 people. The study site is roughly 3 m in elevation and located in the low-country salt marsh region of costal South Carolina^35^. Mean annual temperature is 19.55 °C and mean annual precipitation is 121.51 cm^36^. The area has a year-round population of black vultures and turkey vultures^35^. Major land cover types available to vultures in the study area include evergreen forest (21%), open water (19%), emergent herbaceous wetlands (19%), woody wetlands (9%), developed open space (7%), and grasslands (7%).

Vultures were tagged and monitored to more fully understand their movements and flight behavior in relation to aviation activities at MCAS-Beaufort^29^. Vultures were captured between September 2006 and September 2007 at MCAS-Beaufort using a baited walk-in trap^37^. A uniquely coded white cattle ear tag was attached to the patagium of the right wing on each bird^38,39^. Each bird received a 70-g solar-powered GPS satellite transmitter (PTT-100; Microwave Telemetry Inc., Columbia, MD), attached using a Teflon tape backpack harness^37,40^. Transmitters were attached to 7 turkey vultures (all adults) and 11 black vultures (6 adults and 5 juveniles). Transmitters recorded latitude-longitude (with a 15 m accuracy), altitude above ground level, speed and direction on the hour^29^. Vultures were captured and processed according to procedures specified in study protocol QA-1394, reviewed and approved by the Institutional Animal Care and Use Committee of the National Wildlife Research Center. Attachment of patagial tags and satellite transmitters was authorized under Federal Bird Banding Permit 06859. All methods were performed in accordance with the relevant guidelines and regulations. This study was carried out in compliance with ARRIVE guidelines.

### Roost locations

Transmitters were programmed to operate from dawn to dusk, as determining nighttime roost locations was not a priority in the original telemetry study. Therefore, we inferred night roosting sites from the locations of each vulture two hours before the transmitter stopped in the evening and two hours after it began the next morning. Vultures generally arrive and depart the roost between 2 h before and after sundown and sunrise^16,41^, thus we are confident we captured the actual roosting location using our methods, although we acknowledge the potential for vulture movement at night^42^. We calculated the distance between each successive location for each of these sets of points, choosing the smallest distance moved less than 15 m as the roost location. If the minimum distance moved was greater than 15 m for any set of points, we removed that night from analysis (n = 2216). We conducted separate analyses by species and season, defined as winter (January–March), spring (April–June), summer (July–September), and fall (October–December). In each seasonal analysis, we only included vultures that had at least 15 roost locations. This resulted in removal of three turkey vultures for the fall analysis, one turkey vulture during the summer analysis, and one black vulture during the spring analysis. Some vultures migrated and left the vicinity of the study site, so we limited our analysis to an approximately 65 km radius buffer around MCAS.

### Analysis

We analyzed vulture roost selection using a use-availability framework. The “used” points in this analysis were the roost locations as defined in the previous section. For each of the used roost points, we also identified five paired “available” locations. For each roost point used by a given vulture on a given date, we randomly sampled five actual daily movements, each composed of a distance and a bearing, from the full movement dataset for the corresponding vulture in the corresponding year and season (as defined above). Each pair of movement distance and bearing represented one possible daily movement. Starting at the used roost point, we identified five random available points using these sampled movement distances and bearings. This process was repeated for all used roost points to obtain the paired set of available points.

For each used roost location, we collected values for several covariates. Using data from the 2006 and 2011 National Land Cover Database (NLCD)^43^, we calculated the percent cover of high-density urban (NLCD class 23–24) land use within a 500 m buffer around the roost. We chose this buffer because we were interested in landscape attributes in the vicinity of the roost and vultures have been documented roosting an approximately equivalent distance away from food sources^44^. Additionally, a similarly sized buffer has been used in previous examinations of roosting habitat selection of both species^28^. We used the 2006 NLCD data for 2006–2008 roost locations, and the 2011 NLCD data for the 2009–2012 locations. We also used the NLCD data to calculate habitat type richness within the 500 m buffer. We obtained mean elevation within the buffer from a digital elevation model downloaded from Amazon Terrain Tiles^45^. Using TIGER/Line spatial data^46^, we calculated the total length of roads (m) within the 500 m buffer, as well as the distance (m) from the roost point to the nearest body of water within 5 km (lake or river). We then calculated the same metrics for the set of available points. Correlation between pairs of covariates was < 0.64 in all cases. Since we had a dataset where each used location was paired with a set of available locations (i.e., a strata), we fit conditional logistic regression models (Lehman et al. 2015). We fit separate sets of candidate models for each vulture species (black vulture and turkey vulture) and for each season.

We defined a set of candidate models for each species and season using a two-step process^47^. In the first step, we determined the most informative relationship between the binary response and each covariate: either linear, quadratic, or pseudo-threshold (i.e., natural log). To do this, we fit three univariate models containing only the covariate of interest, one for each transformation. We then ranked these models using QIC^48^ and selected the transformation that minimized the QIC score. We then used the selected transformation in subsequent multivariate models. The set of candidate multivariate models for each species and season was defined as all possible subsets of individual covariates with the appropriate transformation applied to each. All models also included the unique vulture ID as a clustering variable to account for correlated observations of individual vultures.

We ranked the set of candidate models using QIC (Pan 2001), and retained models with ΔQIC ≤ 4 in the set of top models. We did not use model averaging on the set of top models, given the potential issues identified in Cade^49^. All conditional logistic regression models were fit using the “clogit” function in package “survival”^50^ in R version 4.0^51^. We validated the top model(s) with the used-habitat calibration approach of Fieberg, et al.^52^ and calculated concordance as a metric of goodness-of-fit^53^. To determine if variation among individual vultures was influencing our results, we also fit the top-ranked models for each species and season with random slopes as described by^54^ using glmmTMB^55^.

## Results

**Figure 1:**
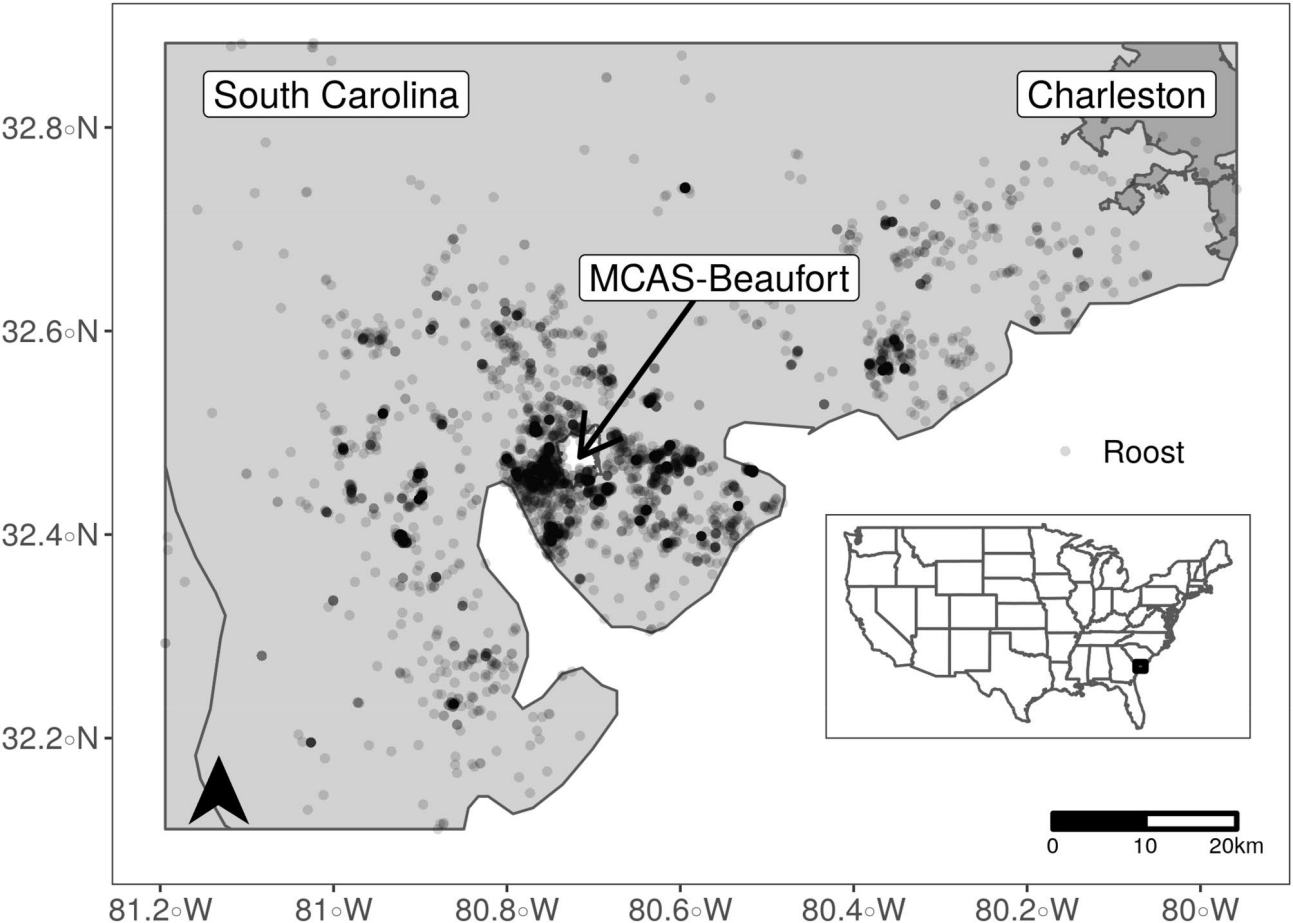
Map of roost locations of 11 black vultures and 7 turkey vultures across southeastern South Carolina, USA. Vultures were tagged at Marine Corps Air Station Beaufort (MCAS-Beaufort) between September 2006 and September 2007. Map was created using packages ggplot2^72^ and sf^73^ in R version 4.0^51^.

We included data from 7 turkey and 11 black vultures in our analysis. Transmission duration averaged 683 d (range 146 to 1702 d), and overall spanned September 2006-May 2012. From these individuals we identified roost locations for a total of 7916 nights (Fig. 1). Roost sample size per individual was similar among seasons and averaged 124 locations per individual per season for turkey vulture (range 17–312) and 112 locations for black vultures (range 20–274). For each combination of vulture species and season, we generally found a single top model with most of the model weight (weight > 0.5; Table 1). Additional models with some support (ΔQIC < 4) generally had similar covariates to the top model (Supplementary Tables S1-S2 online). For the remainder of the results, we focus on the top-ranked model for each species and season combination. Top-ranked models for turkey vultures for all four seasons had mediocre to good fit (concordance ≤ 0.65; Table 1) and were reasonably well-calibrated based on used-habitat calibration (UHC) plots (see Supplementary Figs S1-S4 online). Models for black vultures did not fit as well for most seasons (concordance generally ≤ 0.60; Table 1), but model calibration was adequate (see Supplementary Figs S5-S8 online). Random slopes versions of the top ranked models revealed similar inferences (Supplementary Table S3 online).

**Table 1:** Top-ranked models comparing used and available roost locations by vulture species and season, southern South Carolina, USA, 2006–2012.

Species	Season	Model	Weight	Concordance
TV	Winter	quad(RI) + log(WD) + quad(RL) + HU + quad(EL)	0.60	0.67
TV	Spring	quad(RI) + log(WD) + quad(RL) + quad(EL)	0.51	0.67
TV	Summer	log(RI) + log(WD) + quad(HU)	0.36	0.66
TV	Fall	quad(RI) + log(WD) + quad(RL) + quad(HU)	0.77	0.68
BV	Winter	log(RI) + log(WD) + HU	0.95	0.62
BV	Spring	quad(RI) + log(WD) + quad(HU)	0.56	0.58
BV	Summer	quad(RI) + log(WD) + HU	0.42	0.60
BV	Fall	log(RI) + log(WD) + quad(RL) + quad(EL)	0.49	0.62

Species included were turkey vulture (TV) and black vulture (BV). Model covariates were collected in a 500 m buffer around points and included patch type richness (RI), high-density urban cover (HU), elevation (EL), total road length (RL), and distance to water (WD). Transformations applied to some covariates included quadratic (quad), and natural log (log).

### Land use effects

**Figure 2:**
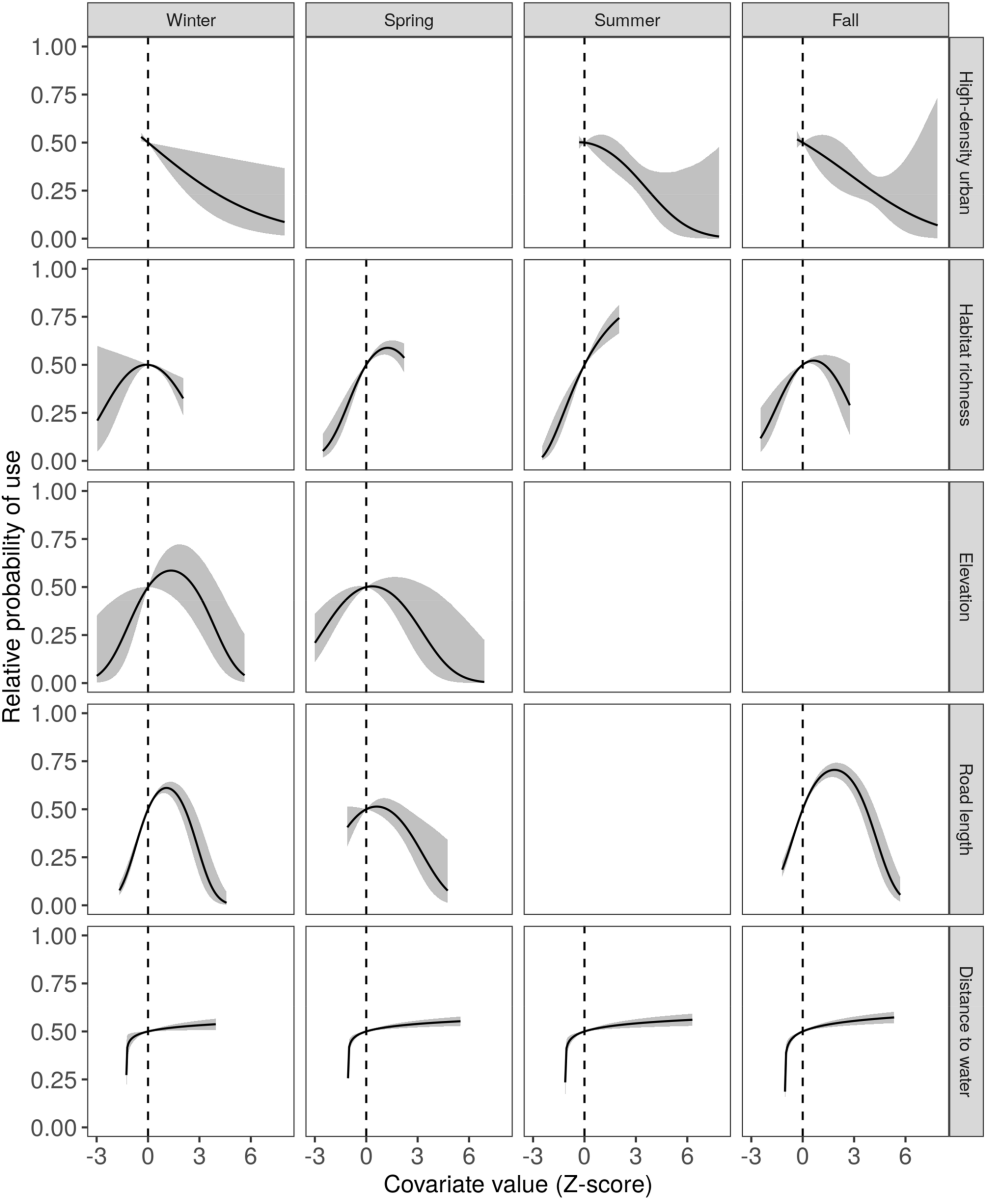
Relative probability of turkey vulture roost site resource selection as a function of covariates (rows) included in the top-ranked model each season (columns). The relative probability represents the probability a vulture would select a site with the given focal covariate value over a second site where the focal covariate value is equal to the mean of that covariate (represented by the dotted line), with all other covariates also held at their mean values. The shaded area represents a 95% confidence envelope around the relative probability. Blank panels in the figure indicate that the corresponding covariate was not in the best model for that season.

**Figure 3:**
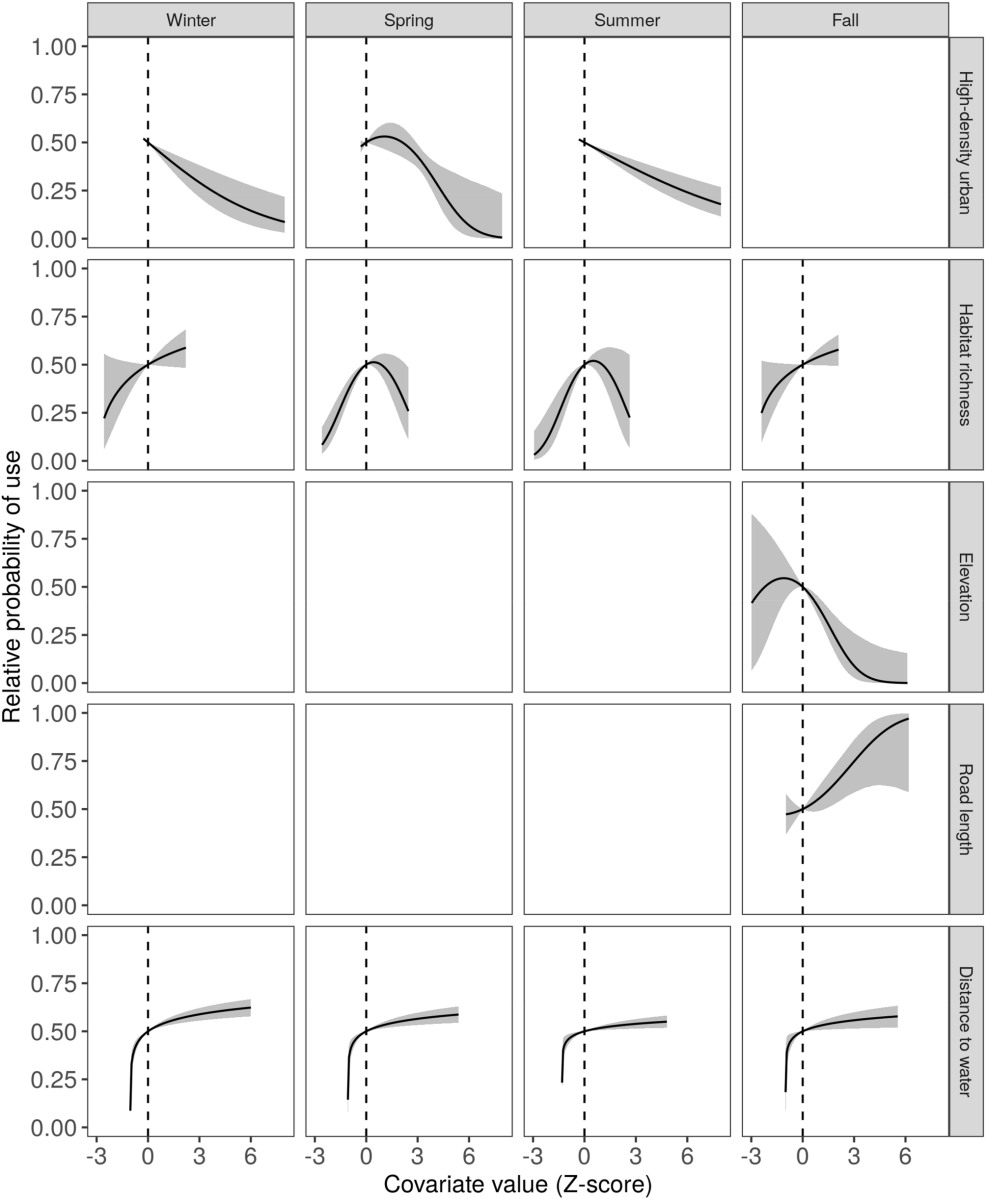
Relative probability of black vulture roost site resource selection as a function of covariates (rows) included in the top-ranked model each season (columns). The relative probability represents the probability a vulture would select a site with the given focal covariate value over a second site where the focal covariate value is equal to the mean of that covariate (represented by the dotted line), with all other covariates also held at their mean values. The shaded area represents a 95% confidence envelope around the relative probability. Blank panels in the figure indicate that the corresponding covariate was not in the best model for that season.

Complete coefficient values and 95% confidence interval for results presented in the next two sections can be found in Supplementary Tables S4–S5 online. High-density urban land cover had a negative impact on turkey vulture roost site use in the winter (β = − 0.30, 95% CI: − 0.47 to − 0.12) and we observed a similar negative relationship in the summer and fall (Fig. 2). Black vulture roost site use also had a negative relationship with high density urban cover in winter (β = − 0.30, 95% CI: − 0.42 to − 0.17) and summer (β = − 0.19, 95% CI − 0.31 to − 0.07) (Fig. 3). Turkey vulture habitat use was positively related to land cover richness in the summer (β = 1.00, 95% CI 0.83–1.18) and maximized at intermediate levels of land cover richness, or 9–11 land cover classes, in winter (β_x_ = 1.08, 95% CI 0.10–2.05; β_x_^2^ = − 0.97, 95% CI − 1.79 to − 0.15), spring (β_x_ = 1.89, 95% CI 1.14 to 2.65; β_x_^2^ = − 1.18, 95% CI − 1.81 to − 0.54), and fall (β_x_ = 1.52, 95% CI 0.76 to 2.29; β_x_^2^ = − 1.12, 95% CI − 1.76 to − 0.48) (Fig. 2). For black vultures, use was maximized at intermediate levels of richness in the spring (β_x_ = 1.83, 95% CI 1.25–2.42; β_x_^2^ = − 1.48, 95% CI − 2.02 to − 0.94) and summer (β_x_ = 2.31, 95% CI 1.63–2.99; β_x_^2^ = − 1.84, 95% CI − 2.45 to − 1.24), and there was a positive relationship with richness, reaching a threshold, in winter (β = 0.30, 95% CI 0.17 to 0.42) and fall (β = 0.28, 95% CI 0.13 to 0.43) (Fig. 3).

### Landscape features

Roost selection was highest at intermediate elevations, or 0–10 m above sea level, in the winter (β_x_ = 4.17, 95% CI 1.01–7.34; β_x_^2^ = − 3.72, 95% CI − 6.77 to − 0.68) and spring (β_x_ = 2.27, 95% CI 0.35–4.19; β_x_^2^ = − 2.36, 95% CI: − 4.27 to − 0.45) for turkey vultures (Fig. 2) and in the fall (β_x_ = 3.45, 95% CI 1.17–5.74; β_x_^2^ = − 3.89, 95% CI − 6.12 to − 1.66) for black vultures (Fig. 3). For turkey vultures, roost selection peaked at intermediate road densities (4.5–10.1 km road length per km^2^ area) in winter (β_x_ = 2.13, 95% CI 1.59–2.67; β_x_^2^ = − 1.52, 95% CI − 1.97 to − 1.08), spring (β_x_ = 0.51, 95% CI 0.20–0.82; β_x_^2^ = − 0.49, 95% CI: − 0.78 to − 0.21), and fall (β_x_ = 1.54, 95% CI: 1.25–1.83; β_x_^2^ = − 1.06, 95% CI: − 1.32 to − 0.79) (Fig. 2). The top-ranked model for black vultures in the fall included a positive relationship with road density (β_x_ = 0.05, 95% CI: − 0.24 to 0.33; β_x_^2^ = 0.21, 95% CI: − 0.06 to 0.48) (Fig. 3). Distance to water had a consistent effect on roost use across species and seasons. In all cases, there was a threshold effect; use was low very close to water and increased with distance from water until reaching a threshold (around 500 m) (Figs. 2–3).

## Discussion

We found support for our hypothesis that anthropogenic landscape elements influenced roost selection by turkey vultures and black vultures. In agreement with our prediction, roosting by both species was positively associated with roads to some degree^56^. The peak of turkey vulture roosting at intermediate road densities may represent a tradeoff between resource availability with the risk of vehicle collision, which is likely elevated at higher road densities^57^. This may also account for the lack of relationship with roads during summer when seasonal tourism results in more vehicle traffic. Similarly, use of roads by cinereous vultures (*Aegypius monachus*) and griffon vultures (*Gyps fulvus*) declined at high traffic volumes^58^. The response of black vultures to roads was also positive in one season, but did not decline at high road densities. Black vultures tend to exhibit bolder behavior than turkey vultures, especially when in groups^28,59^, which could make them less sensitive to high traffic levels and greater human presence compared to turkey vultures. The positive selection of roads by both species suggests that growth of the road network will be favorable to some extent for both turkey and black vultures.

Our prediction that roosting would be influenced by landscape fragmentation was also supported for both species. The positive response may result from increases in obstruction currents, which facilitate flight, as landscapes become fragmented^18^. Additionally, as habitat richness increases, there are more contrasting surface temperatures resulting from different land cover types, which creates stronger thermal currents^23^. Heterogeneity in habitats can also increase the resources available for vultures such as food, water, and perching sites^23^. However, at high levels of richness, there may be more habitats intensely used by humans, which could account for the decline in roosting at high values of habitat richness in some seasons.

In contrast to our predictions, vultures generally either selected against or showed no response to urban land cover. There may be differences in urban development across study areas that influence resource availability for vultures. In urban Brazil, for example, vultures often feed at large open street markets, which do not exist in Beaufort^24^. Differences in garbage management also influence feeding opportunities for vultures in urban areas^60^. Furthermore, vultures may select for certain types of buildings such as skyscrapers that are absent in our study area^61^. It is also plausible that although both species can survive in urban areas, such habitat is not their preference. With Beaufort being a relatively small town, urban cover is not extensive and vultures may avoid it due to the availability of alternative habitats that are more suitable.

Both species avoided roosting locations very close (< 500 m) to water, which was also contrary to our predictions and in opposition to previous work^18,19^. Vulture roosts in the Beaufort area included a municipal water tower (630 m from water) and a communication tower (750 m from water), (J. Humphrey, unpublished data), and environmental guidelines often recommend such structures be placed a minimum distance from water sources e.g.^62^. As a result, relationships between roost selection and water may reflect vulture selection for towers rather than selection against water. A similar association may account for the selection of roosting at intermediate elevation values for both species, which also contradicted our prediction. Peaks of roosting were at relatively low elevations (less than 10 m), which would not result in the degree of terrain ruggedness required to produce strong currents e.g.^63^. Elevation is thus probably correlated with some other quality of the landscape for which our analysis does not account.

We found no evidence of pronounced seasonal differences in the relationship between landscape variables and roosting probability for either species. Roost site selection by turkey vultures only differed by one variable (elevation) between fall and winter, the seasons when they show the largest and smallest home ranges, respectively^29^. For black vultures, variables influencing probability of roosting were identical between their seasons of largest and smallest home ranges (summer and winter). Additionally, the relationship between roosting and landscape features tended to be the same across seasons. These results suggest that there is seasonal consistency in the influence of landscape features on vulture roosting probability, regardless of fluctuations in home range size and space use.

Our models had greater predictive ability for determining roost locations of turkey vultures compared to black vultures, as measured by concordance values. Additionally, turkey vulture roost selection was more likely to be influenced by the variables we assessed. Interspecific differences in foraging strategies and sociality likely account for these differences. Turkey vultures primarily locate carrion using olfaction, whereas black vultures rely on visual cues and the presence of other foraging vultures to detect carcasses^64–66^. Communal roosts may function as information centers for black vultures; by following individuals with knowledge of food sources when departing roosts, naïve black vultures can increase foraging success^67,68^. Turkey vultures, by contrast, are less likely to use communal roosts as centers of information exchange^69^. As a result, black vulture roost site selection may be influenced by the presence of conspecifics in addition to landscape attributes. Furthermore, black vultures tend to reuse roosts, whereas turkey vultures are more apt to use different roosts from night to night^28^. Thus, there is a greater chance that turkey vultures select roosts based on favorable conditions, rather than returning to previously used roosts. This divergence in roost fidelity may have also contributed to the lower number of landscape features influencing black vulture roosting and account for our limited ability to predict black vulture roosting locations based on landscape elements.

Comparisons with previous work indicate variability in black and turkey vulture roosting habitat selection, even across relatively small spatial scales. At the Savannah River Site (SRS), located 150 km from our study area, both species tended to roost further from roads, whereas road density had a positive effect on vultures in our study area for most of the year^28^. Taken alongside the divergent responses to urban cover and water previously described, these findings imply a high degree of flexibility in roosting habitat selection of black and turkey vultures. As a result, we warrant caution for extrapolating roost site selection findings outside of specific study areas. The success of other birds, such as European starlings (*Sturnus vulgaris*), in human-modified landscapes has been attributed partially to roost selection flexibility^8^, and our results suggest the same may be true for turkey vultures and black vultures.

On the whole, our results indicate that anthropogenic disturbance may benefit turkey vultures and black vultures in terms of roosting habitat availability. These findings are in accordance with previous work suggesting that turkey vultures are one of the bird species benefitting most from an increase in the growth of small towns^70^. Despite the important ecosystem services provided by vultures, expansions in human development can be expected to entail an increase in human-vulture conflict through property damage and human health concerns^30^. Both species are also frequently involved in aircraft collisions, with turkey vultures ranking as the third riskiest bird species to civil aircraft across the United States^71^. However, the versatility in habitat selection limits the ability to make reliable inferences in roost-site selection across large spatial scales. For black vultures, this is further complicated by the influence of conspecifics and social interactions on roosting habitat selection. As a result, site-specific investigations of roost-site selection will likely be required to effectively manage vulture populations and mitigate human-vulture conflict as natural landscapes are increasingly impacted by human development.

## Supplementary Information

Supplementary Information.
