# Supplementary material for: Landscape transformations produce favorable roosting conditions for turkey vultures and black vultures
Source: Sci Rep. 2021 Jul 20;11:14793. doi: 10.1038/s41598-021-94045-3 (PMC8292396; doi:10.1038/s41598-021-94045-3)

Supplementary Information

Supplementary tables and figures for:

Jacob E. Hill, Kenneth F. Kellner, Bryan M. Kluever, Michael L. Avery, John S. Humphrey, Eric A. Tillman, Travis L. DeVault, and Jerrold L. Belant. 2021. Landscape transformations produce favorable roosting conditions for turkey vultures and black vultures.

# Tables

**Table S1.** Model selection results comparing used and available roost locations for turkey vultures in southern South Carolina, USA, 2006-2012. Models were fit and selected independently for each season. Models were ranked according to QIC. Models with ΔQIC ≤ 4 are shown for each season. If fewer than three models met this criteria then the top three models regardless of ΔQIC are shown. The number of parameters in the model (K) and the model weight are also provided. Model covariates were collected in a 500 m buffer around points and included patch type richness (RI), high-density urban cover (HU), elevation (EL), total road length (RL), and distance to water (WD). Transformations applied to some covariates included quadratic (quad), and natural log (log).

| Season | Model | QIC | ΔQIC | K | weight | concord |
| --- | --- | --- | --- | --- | --- | --- |
| Winter | quad(RI)+log(WD)+quad(RL)+HU+quad(EL) | 2182.59 | 0.00 | 8 | 0.60 | 0.67 |
| Winter | log(WD)+quad(RL)+HU+quad(EL) | 2184.72 | 2.13 | 6 | 0.21 | 0.65 |
| Winter | quad(RI)+quad(RL)+HU+quad(EL) | 2185.80 | 3.21 | 7 | 0.12 | 0.67 |
| Spring | quad(RI)+log(WD)+quad(RL)+quad(EL) | 2522.99 | 0.00 | 7 | 0.51 | 0.67 |
| Spring | quad(RI)+log(WD)+quad(RL)+log(HU)+quad(EL) | 2525.00 | 2.01 | 8 | 0.19 | 0.67 |
| Spring | quad(RI)+log(WD)+quad(RL) | 2526.38 | 3.39 | 5 | 0.09 | 0.66 |
| Summer | log(RI)+log(WD)+quad(HU) | 2924.21 | 0.00 | 4 | 0.36 | 0.66 |
| Summer | log(RI)+log(WD)+quad(RL)+quad(HU) | 2924.73 | 0.53 | 6 | 0.28 | 0.66 |
| Summer | log(RI)+log(WD)+quad(RL)+quad(HU)+quad(EL) | 2925.07 | 0.86 | 8 | 0.24 | 0.66 |
| Summer | log(RI)+log(WD)+quad(HU)+quad(EL) | 2926.41 | 2.20 | 6 | 0.12 | 0.66 |
| Fall | quad(RI)+log(WD)+quad(RL)+quad(HU) | 2934.96 | 0.00 | 7 | 0.77 | 0.68 |
| Fall | quad(RI)+log(WD)+quad(RL)+quad(HU)+quad(EL) | 2937.35 | 2.39 | 9 | 0.23 | 0.68 |
| Fall | quad(RI)+log(WD)+quad(RL) | 2953.13 | 18.17 | 5 | 0.00 | 0.67 |

**Table S2.** Model selection results comparing used and available roost locations for black vultures in southern South Carolina, USA, 2006-2012. Models were fit and selected independently for each season. Models were ranked according to QIC. Models with ΔQIC ≤ 4 are shown for each season. If fewer than 3 models met this criteria then the top three models regardless of ΔQIC are shown. The number of parameters in the model (K) and the model weight are also provided. Model covariates were collected in a 500 m buffer around points and included patch type richness (RI), high-density urban cover (HU), elevation (EL), total road length (RL), and distance to water (WD). Transformations applied to some covariates included quadratic (quad), and natural log (log).

| Season | Model | QIC | ΔQIC | K | weight | concord |
| --- | --- | --- | --- | --- | --- | --- |
| Winter | log(RI)+log(WD)+HU | 3915.62 | 0.00 | 3 | 0.95 | 0.62 |
| Winter | log(WD)+HU | 3922.01 | 6.39 | 2 | 0.04 | 0.62 |
| Winter | log(RI)+log(WD)+RL+HU+quad(EL) | 3924.68 | 9.06 | 6 | 0.01 | 0.63 |
| Spring | quad(RI)+log(WD)+quad(HU) | 3699.65 | 0.00 | 5 | 0.56 | 0.58 |
| Spring | quad(RI)+log(WD)+quad(HU)+quad(EL) | 3701.74 | 2.09 | 7 | 0.20 | 0.58 |
| Spring | quad(RI)+log(WD)+RL+quad(HU)+quad(EL) | 3701.94 | 2.29 | 8 | 0.18 | 0.59 |
| Summer | quad(RI)+log(WD)+HU | 3897.26 | 0.00 | 4 | 0.42 | 0.60 |
| Summer | quad(RI)+log(WD)+RL+HU+quad(EL) | 3897.69 | 0.42 | 7 | 0.34 | 0.60 |
| Summer | quad(RI)+log(WD)+RL+HU | 3899.20 | 1.93 | 5 | 0.16 | 0.60 |
| Summer | quad(RI)+log(WD)+HU+quad(EL) | 3900.60 | 3.34 | 6 | 0.08 | 0.61 |
| Fall | log(RI)+log(WD)+quad(RL)+quad(EL) | 4172.97 | 0.00 | 6 | 0.49 | 0.62 |
| Fall | log(RI)+log(WD)+quad(RL)+HU+quad(EL) | 4173.02 | 0.05 | 7 | 0.48 | 0.61 |
| Fall | log(WD)+quad(RL)+quad(EL) | 4180.60 | 7.63 | 5 | 0.01 | 0.61 |

**Table S3.** Comparison between parameter estimates from fixed and random-slope versions of top-ranked models by species (TV = turkey vulture, BV = black vulture) and season. Model covariates included patch type richness (RI), high-density urban cover (HU), elevation (EL), total road length (RL), and distance to water (WD).

| Species | Season | Parameter | Fixed | Random |
| --- | --- | --- | --- | --- |
| TV | Winter | RI | 1.08 | 1.25 |
| TV | Winter | RI^2 | -0.97 | -1.09 |
| TV | Winter | log(WD) | 0.25 | 0.23 |
| TV | Winter | RL | 2.13 | 2.27 |
| TV | Winter | RL^2 | -1.52 | -1.58 |
| TV | Winter | HU | -0.30 | -0.44 |
| TV | Winter | EL | 4.17 | 6.02 |
| TV | Winter | EL^2 | -3.72 | -5.98 |
| TV | Spring | RI | 1.89 | 2.35 |
| TV | Spring | RI^2 | -1.18 | -1.62 |
| TV | Spring | log(WD) | 0.31 | 0.29 |
| TV | Spring | RL | 0.51 | 0.74 |
| TV | Spring | RL^2 | -0.49 | -0.70 |
| TV | Spring | EL | 2.27 | 3.35 |
| TV | Spring | EL^2 | -2.36 | -3.78 |
| TV | Summer | log(RI) | 1.00 | 1.13 |
| TV | Summer | log(WD) | 0.35 | 0.39 |
| TV | Summer | HU | 0.00 | -0.09 |
| TV | Summer | HU^2 | -0.50 | -0.38 |
| TV | Fall | RI | 1.52 | 1.53 |
| TV | Fall | RI^2 | -1.12 | -1.15 |
| TV | Fall | log(WD) | 0.44 | 0.44 |
| TV | Fall | RL | 1.54 | 1.62 |
| TV | Fall | RL^2 | -1.06 | -1.18 |
| TV | Fall | HU | -0.20 | -0.19 |
| TV | Fall | HU^2 | -0.13 | -0.13 |
| BV | Winter | log(RI) | 0.30 | 0.57 |
| BV | Winter | log(WD) | 0.65 | 0.82 |
| BV | Winter | HU | -0.30 | -0.35 |
| BV | Spring | RI | 1.83 | 2.01 |
| BV | Spring | RI^2 | -1.48 | -1.62 |
| BV | Spring | log(WD) | 0.52 | 0.58 |
| BV | Spring | HU | 0.31 | 0.31 |
| BV | Spring | HU^2 | -1.29 | -1.17 |
| BV | Summer | RI | 2.31 | 2.04 |
| BV | Summer | RI^2 | -1.84 | -1.63 |
| BV | Summer | log(WD) | 0.29 | 0.55 |
| BV | Summer | HU | -0.19 | -0.18 |
| BV | Fall | log(RI) | 0.28 | 0.45 |
| BV | Fall | log(WD) | 0.41 | 0.53 |
| BV | Fall | RL | 0.05 | 0.19 |
| BV | Fall | RL^2 | 0.21 | -0.02 |
| BV | Fall | EL | 3.45 | 4.69 |
| BV | Fall | EL^2 | -3.89 | -5.02 |

**Table S4**. Parameter estimates and 95% confidence intervals from the top-ranked model of habitat use for turkey vultures in each season.

| Parameter | Winter | | | Spring | | | Summer | | | Fall | | |
| --- | --- | --- | --- | --- | --- | --- | --- | --- | --- | --- | --- | --- |
|  | Estimate | Lower | Upper | Estimate | Lower | Upper | Estimate | Lower | Upper | Estimate | Lower | Upper |
| EL | 4.17 | 1.01 | 7.34 | 2.27 | 0.35 | 4.19 |  |  |  |  |  |  |
| EL^2 | -3.72 | -6.77 | -0.68 | -2.36 | -4.27 | -0.45 |  |  |  |  |  |  |
| HU | -0.30 | -0.47 | -0.12 |  |  |  | 0.00 | -0.29 | 0.29 | -0.20 | -0.48 | 0.08 |
| HU^2 |  |  |  |  |  |  | -0.50 | -1.04 | 0.05 | -0.13 | -0.63 | 0.36 |
| RI | 1.08 | 0.10 | 2.05 | 1.89 | 1.14 | 2.65 |  |  |  | 1.52 | 0.76 | 2.29 |
| RI^2 | -0.97 | -1.79 | -0.15 | -1.18 | -1.81 | -0.54 |  |  |  | -1.12 | -1.76 | -0.48 |
| log(RI) |  |  |  |  |  |  | 1.00 | 0.83 | 1.18 |  |  |  |
| RL | 2.13 | 1.59 | 2.67 | 0.51 | 0.20 | 0.82 |  |  |  | 1.54 | 1.25 | 1.83 |
| RL^2 | -1.52 | -1.97 | -1.08 | -0.49 | -0.78 | -0.21 |  |  |  | -1.06 | -1.32 | -0.79 |
| log(WD) | 0.25 | 0.00 | 0.50 | 0.31 | 0.15 | 0.48 | 0.35 | 0.20 | 0.49 | 0.44 | 0.26 | 0.62 |

**Table S5**. Parameter estimates and 95% confidence intervals from the top-ranked model of habitat use for black vultures in each season.

| Parameter | Winter | | | Spring | | | Summer | | | Fall | | |
| --- | --- | --- | --- | --- | --- | --- | --- | --- | --- | --- | --- | --- |
|  | Estimate | Lower | Upper | Estimate | Lower | Upper | Estimate | Lower | Upper | Estimate | Lower | Upper |
| EL |  |  |  |  |  |  |  |  |  | 3.45 | 1.17 | 5.74 |
| EL^2 |  |  |  |  |  |  |  |  |  | -3.89 | -6.12 | -1.66 |
| HU | -0.30 | -0.42 | -0.17 | 0.31 | -0.03 | 0.64 | -0.19 | -0.31 | -0.07 |  |  |  |
| HU^2 |  |  |  | -1.29 | -2.30 | -0.28 |  |  |  |  |  |  |
| RI |  |  |  | 1.83 | 1.25 | 2.42 | 2.31 | 1.63 | 2.99 |  |  |  |
| RI^2 |  |  |  | -1.48 | -2.02 | -0.94 | -1.84 | -2.45 | -1.24 |  |  |  |
| log(RI) | 0.30 | 0.17 | 0.42 |  |  |  |  |  |  | 0.28 | 0.13 | 0.43 |
| RL |  |  |  |  |  |  |  |  |  | 0.05 | -0.24 | 0.33 |
| RL^2 |  |  |  |  |  |  |  |  |  | 0.21 | -0.06 | 0.48 |
| log(WD) | 0.65 | 0.50 | 0.81 | 0.52 | 0.37 | 0.67 | 0.29 | 0.16 | 0.41 | 0.41 | 0.28 | 0.55 |

# Figures

Used-habitat calibration plots for the top-ranked models by vulture species and season. For each top model, data were split into training (75%) and testing (25%) sets. The model was fit with the training set and a predictive distribution of use based on a range of values for each covariate was simulated using the testing dataset. In each panel in the figures below, the shaded gray area represent the 95% simulation envelope. The blue line represents the used distribution of the covariate. If the model is well-calibrated, the blue line should fall inside the shaded area. The red line is the distribution of covariate values available on the landscape.

**Figure S1**. Used-habitat calibration plot for the top-ranked model of turkey vulture habitat use in the winter.


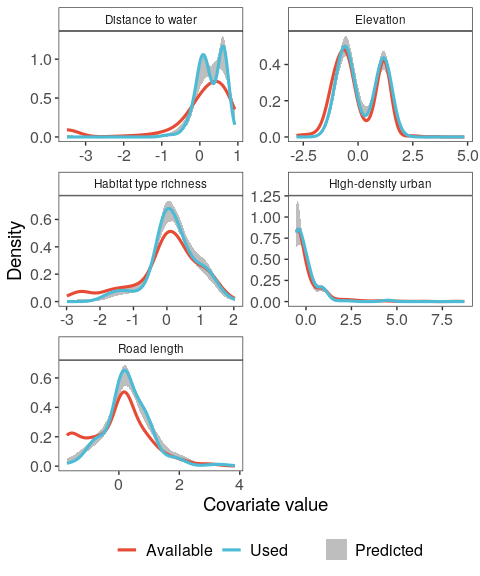


**Figure S2**. Used-habitat calibration plot for the top-ranked model of turkey vulture habitat use in the spring.


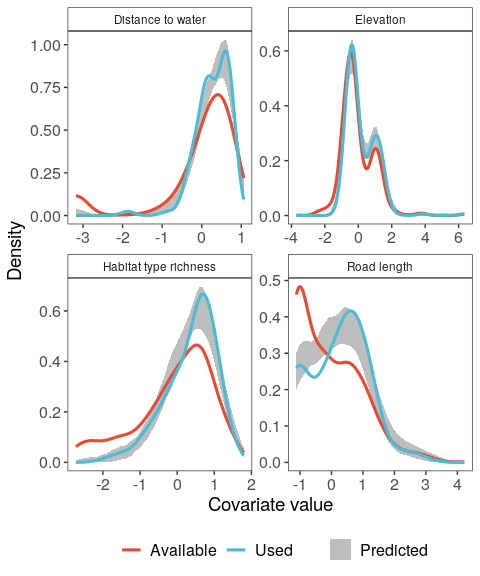


**Figure S3**. Used-habitat calibration plot for the top-ranked model of turkey vulture habitat use in the summer.


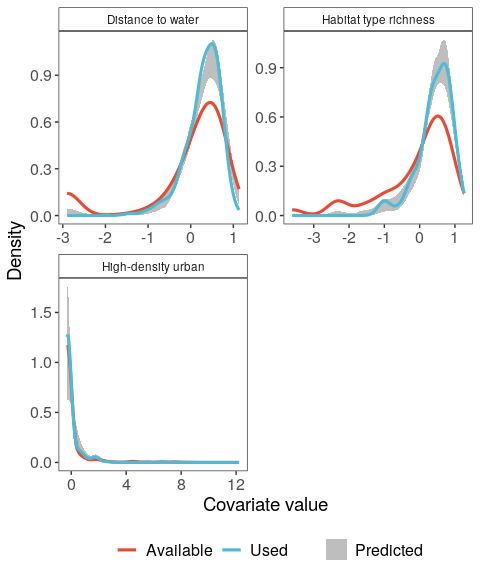


**Figure S4**. Used-habitat calibration plot for the top-ranked model of turkey vulture habitat use in the fall.


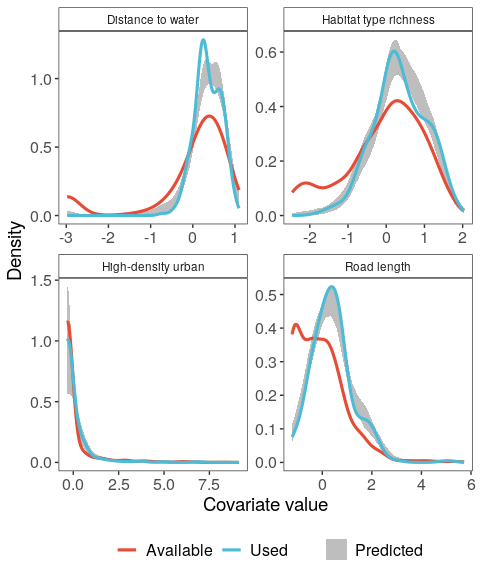


**Figure S5**. Used-habitat calibration plot for the top-ranked model of black vulture habitat use in the winter.


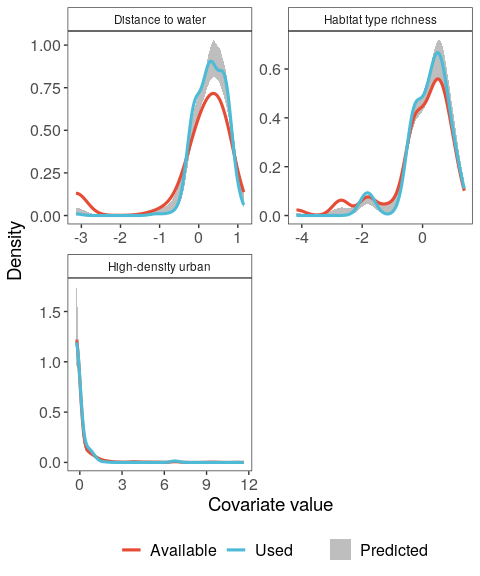


**Figure S6**. Used-habitat calibration plot for the top-ranked model of black vulture habitat use in the spring.


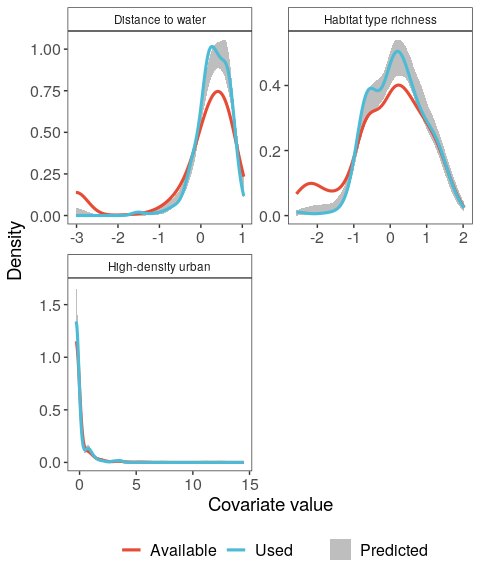


**Figure S7**. Used-habitat calibration plot for the top-ranked model of black vulture habitat use in the summer.


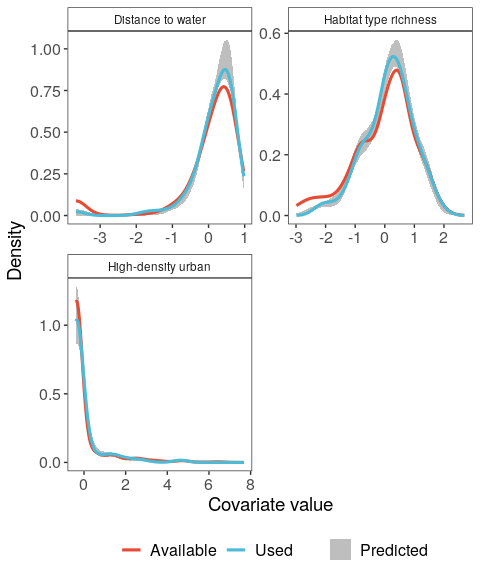


**Figure S8**. Used-habitat calibration plot for the top-ranked model of black vulture habitat use in the fall.


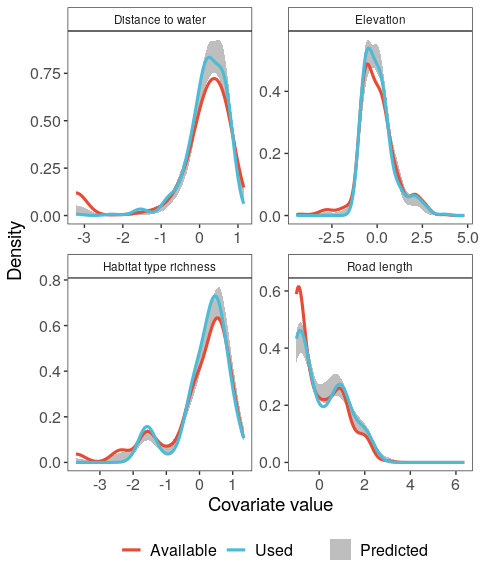

Supplement: Supplementary file 1 — Supplementary Information. [file 41598_2021_94045_MOESM1_ESM.docx]
